# Effect of knee flexion on muscle oxygen saturation in adults during a 40‐s isometric squat

**DOI:** 10.14814/phy2.70808

**Published:** 2026-03-11

**Authors:** Enmanuel Portilla‐Dorado, Carlos Sendra‐Perez, Joaquín Martín Marzano‐Felisatti, Inmaculada Aparicio‐Aparicio, Rosa M. Cibrián Ortiz de Anda, Jose I. Priego‐Quesada

**Affiliations:** ^1^ Universidad del Cauca Popayán Cauca Colombia; ^2^ Research Group in Sports Biomechanics (GIBD), Department of Physical Education and Sports Universitat de València Valencia Spain; ^3^ Department of Education and Specific Didactics Jaume I University Castellon Spain; ^4^ Research Group in Medical Physics (GIFIME), Department of Physiology University of Valencia Valencia Spain

**Keywords:** EMG, kinematics, muscle excitation, NIRS, strength

## Abstract

Monitoring internal load is essential to understanding fatigue and training adaptation in athletes. This study aimed to evaluate the effect of different knee flexion angles during a 40‐s isometric squat on muscle oxygen saturation (SmO_2_) responses. Seventeen physically active participants (9 males and 8 females: 28 ± 9 years old, 174 ± 12 cm, and 70.1 ± 16.6 kg) performed three randomized squats at 80°, 90°, and 100° knee angles. Muscle excitation (i.e., RMS) was measured using surface electromyography (EMG), and SmO_2_ was recorded with near‐infrared spectroscopy technology. Results indicated that SmO_2_ differed only between the most extreme angles (80° vs. 100°), with significantly greater deoxygenation at 80°, emerging in the final 20% of the squat (*p* < 0.01 and 95% CI of the differences [6, 21%]). In contrast, the RMS of the squat at 80° was higher than at 90° (*p* = 0.03 and 95% CI [0.4, 8.5%]) and at 100° (*p* < 0.001 and 95% CI [5.8, 14.2%]). The findings suggest that 90° knee flexion elicits an intermediate metabolic and muscular excitation response. A 40‐s isometric squat is sufficient to reveal condition‐specific differences in SmO_2_, though differences only emerge toward the end of the task. Thus, using a goniometer for posture control may be sufficient in field settings, offering a practical approach for load monitoring and fatigue assessment during isometric strength testing.

## 1 INTRODUCTION

Monitoring internal load response to physical exertion is essential to understanding an athlete's fatigue status and training adaptation (Gabbett & Oetter, 2025; Halson, 2014). Such monitoring can assist coaches in preventing injury risk and optimizing training design (Mujika, 2017; Soligard et al., 2016). For quantification of internal load, parameters that are commonly used are creatine kinase, blood lactate concentration, muscle activation, heart rate, perception of exertion, delayed muscle onset soreness, and sleep quality, among others (Gabbett & Oetter, 2025; Halson, 2014; Heishman et al., 2018; Lima‐Alves et al., 2021). However, all these methods often present limitations such as high inter‐ and intra‐individual variability, elevated cost, or lengthy data acquisition and processing times (Halson, 2014; Saw et al., 2016). For this reason, it remains a challenge to develop valid and cost‐effective athletic monitoring devices to assess load‐response (Gabbett & Oetter, 2025). Interest in using portable near‐infrared spectroscopy (NIRS) to monitor oxygen availability in the muscle has increased due to the availability of compact, wearable, and affordable commercial devices (Perrey et al., 2024; Vasquez‐Bonilla et al., 2024), and several studies support its use as a tool for assessing internal load (Vasquez‐Bonilla et al., 2024; Yeap et al., 2024; Yogev et al., 2023).

Given that oxygen availability is a key determinant of ATP production and fatigue development (Kime et al., 2003; Perrey et al., 2024), NIRS‐derived muscle oxygen saturation (SmO_2_), which represents the balance between muscle oxygen supply and the metabolic oxygen demand, has become a relevant parameter (Boone et al., 2016; Vasquez‐Bonilla et al., 2024). For this reason, it is being increasingly applied in sports science to evaluate muscle metabolic responses (Perrey et al., 2024; Sendra‐Pérez et al., 2023) and provide the capacity to determine whether oxygen delivery is appropriately matched to metabolic demand (Boone et al., 2016; Feldmann et al., 2020). Previous studies have evaluated the effects of fatigue on SmO_2_ during occlusion tests (Greaves et al., 2024; Keller et al., 2023) or the agreement in oxygen kinetics with phosphocreatine dynamics both in occlusions and isometric contractions (Maliszewski et al., 2024). However, while vascular occlusion tests are technically feasible in field environments, they may be less practical in applied sport settings due to the additional time, discomfort, and nonfunctional nature of the protocol. The results of previous studies suggest that an isometric squat may be a valid test to detect muscle fatigue (Bazyler et al., 2015; Gómez‐Carmona et al., 2020; Lin et al., 2024; Sendra‐Pérez, Marzano‐Felisatti, et al., 2025). Moreover, a previous study showed how the SmO_2_ differences between an exercised and non‐exercised leg are evident between the second 30 and 45 of an isometric quadriceps contraction at 90° of knee flexion (Sendra‐Pérez, Marzano‐Felisatti, et al., 2025). However, it is still unclear whether SmO_2_ is sensitive to postural factors such as knee flexion during functional multi‐joint tasks. Although a previous study demonstrated that oxygen consumption of the knee extensors varies with joint angle during isometric knee extension (30°, 60°, and 90°), their protocol involved single‐joint dynamometer testing rather than a multi‐joint, weight‐bearing task such as the isometric squat (de Ruiter et al., 2005). Moreover, their study focused on relatively large angular differences, whereas it remains unknown whether small, practically relevant deviations in knee angle within a squat posture meaningfully affect SmO_2_ responses. If SmO_2_ were sensitive to small variations in joint angle, it would imply the need for precise control of posture (e.g., via 3D motion capture) when conducting field assessments.

Knee flexion angle during isometric squat exercises has been shown to significantly affect quadriceps muscle excitation and torque output (Kukić et al., 2022; Marchetti et al., 2016; Palmer et al., 2018). The 90° knee flexion position is commonly used in both research and applied settings, as it represents a mechanically stable and functionally relevant posture that allows substantial quadriceps excitation and torque production (Kukić et al., 2022; Marchetti et al., 2016; Sendra‐Pérez, Marzano‐Felisatti, et al., 2025). Marchetti et al. (2016) reported that muscle excitation of different muscles of the quadriceps was greater at 90° of knee flexion compared to other extreme options (i.e., 20° and 140°). In addition, Kukić et al. (2022) also observed that while quadriceps muscle excitation remained relatively stable across knee angles between 80° and 130°, torque output varied significantly, indicating that mechanical leverage rather than neural drive may account for strength differences at higher degrees of flexion. Similarly, Trindade et al. (2020) observed lower vastus lateralis excitation at 60° compared to 90° and 120°, despite maximal strength being highest at 60°. All these results highlight the importance of assessing muscle excitation alongside NIRS measurements to better understand how knee flexion angle may influence SmO_2_ sensitivity.

The objective of this study was to assess the effect of the knee flexion during a 40‐s isometric squat on the SmO_2_ response. It was hypothesized that changes of 10° in knee flexion would not affect SmO_2_, while changes of 20° would result in detectable differences, aligned with variations in muscle excitation. We selected angles ±10° from the 90° reference position to examine whether relatively small and practically feasible deviations, those that could occur during field assessments, would meaningfully influence SmO_2_ responses.

## 2 MATERIALS AND METHODS

### 2.1 Participants

Based on pilot data examining SmO_2_ differences at the end of the 40‐s isometric squat across the three knee flexion angles, an a priori sample size calculation was performed using G*Power 3 (University of Düsseldorf, Düsseldorf, Germany). For a repeated‐measures ANOVA, assuming a large effect size (Cohen's *f* = 0.5), an *α* level of 0.05, a statistical power of 0.90, three repeated measurements, and a correlation among repeated measures of 0.5, the required sample size was estimated at 11 participants. Then, we recruited 17 physically active participants (9 males and 8 females, 28 ± 9 years old, 174 ± 12 cm, and 70.1 ± 16.6 kg, 19.9 ± 6.0% of body fat, and 19 ± 7 mm of anterior thigh skinfold). For the recruitment, an online questionnaire was disseminated to social media and by emails to the Bachelor of Physical Activity and Sports Sciences students of the University of Valencia with information about the study. Inclusion criteria consisted of being healthy (any disease diagnosed or musculoskeletal injury in the last 6 months), aged 18–50 years old and capable of performing a 40‐s isometric squat. Exclusion criteria included smokers, those diagnosed with chronic disease, those taking medications known to influence cardiorespiratory function, or those unable to provide written informed consent. All participants signed the consent form before starting the study. The project was in agreement with the Declaration of Helsinki and was approved by the Ethics Committee of Research in Humans of the University of Valencia (reference 2024‐FIS‐3694985). Participants refrained from exercise on the day of the test.

### 2.2 Protocol

**FIGURE 1:**
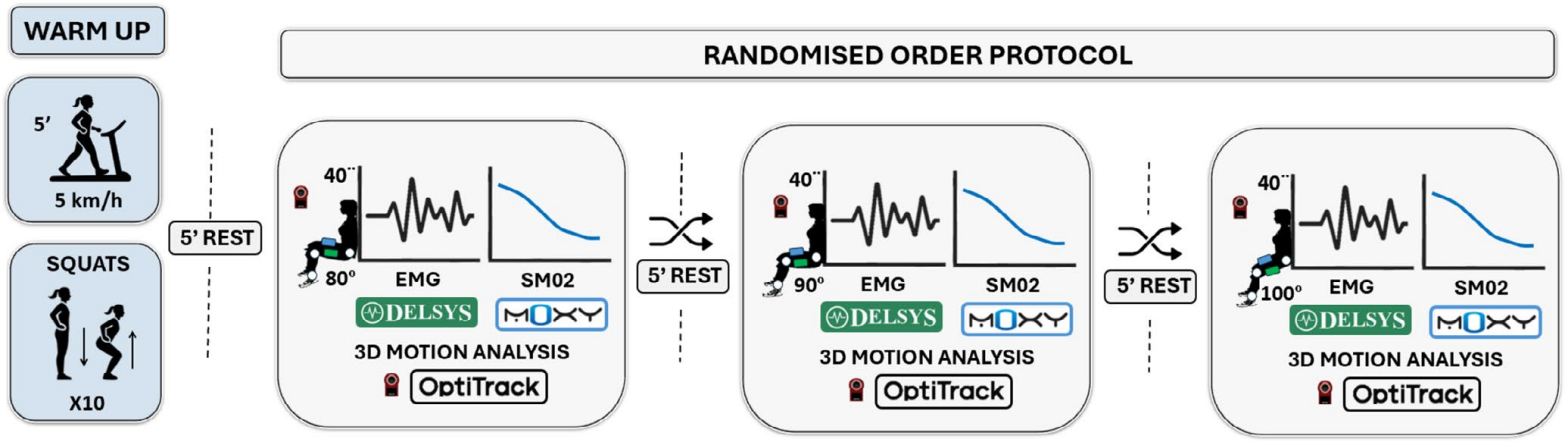
Schematic representation of the study protocol.

Figure 1 shows a schematic representation of the protocol. Participants performed a warm‐up of walking for 5‐min at 5 km·h^−1^ and 10 squats with their own body mass. After this warming‐up, they rested for 5‐min, and then they performed three 40‐s isometric squats, with 5‐min of seated rest between them. Squats were performed with different knee flexions (80°, 90°, and 100°) in a randomized order.

During the squat, participants were required to maintain a static position with their feet shoulder‐width apart, had to look straight ahead, their arms crossed over their chest, their backs touching a wall but not their buttocks, and the soles of their feet touching the floor. These instructions were provided verbally, and the researcher visually ensured that all participants followed them during the squat.

The environmental temperature of the laboratory was set using an air conditioning system to 23°C. Knee flexion was determined by 3D motion analysis, SmO_2_ by NIRS, and muscular excitation by surface electromyography (EMG).

### 2.3 Measurements

Firstly, the body fat percentage of each participant was measured using a bioelectrical impedance system (Tanita RD545, Tanita Corp., Japan). Moreover, skinfolds from the anterior thigh of both lower limbs were assessed by the same researcher using a skinfold caliper (Innovare–Cescorf, Cescorf, Porto Alegre, Brazil) following the protocol established by the International Society of the Advancement of Kinanthropometry (ISAK) for anthropometric assessments. This data was used to characterize the sample.

Knee flexion was indicated by a researcher who, using a manual goniometer of 360°, helped him position himself during knee flexion from a standing position with his legs stretched out. The onset of the 40‐s isometric squat was defined as the moment participants initiated the descent from the standing position. Participants were instructed to reach the target knee angle as quickly as possible (typically within the first 5 s, including adjustments asked by the researcher with the goniometer) and then maintain this position for the remainder of the trial. During the isometric squat, a motion capture system was used (Optitrack, NaturalPoint Inc., Corvallis, OR, USA) for the 3D kinematic analysis using 6 infrared cameras (100 Hz; Flex 3) arranged around the place where the participant performed the squat. This data was used to ensure that the target knee flexion (80°, 90°, or 100°) was performed. The OptiTrack motion capture system was calibrated before each testing session, following manufacturer guidelines (i.e., wand and ground plane calibrations). The mean three‐dimensional reconstruction error after calibration was between 3 and 5 mm. Data was acquired from the cameras through Motive tracker software (version 1.10, NaturalPoint Inc., OR, USA). Reflective markers to measure knee flexion were attached to the lateral malleolus, the lateral femoral condyle, and the greater trochanter of both lower limbs. These markers defined two segments (thigh and shank) for each lower limb, and knee flexion angle was calculated by the projected β angle between these two segments. The knee flexion of each lower limb was averaged to obtain a mean knee flexion during the isometric squat. Moreover, this knee flexion was corrected with the flexion obtained during the anatomic standing position (considering that the knee was not flexed and its real angle is 0°).

The SmO_2_ was measured using NIRS technology (Moxy Monitor, Fortiori Design LLC, Minneapolis, USA). As EMG and NIRS devices were both located on the vastus lateralis, following surface electromyography guidelines (Hermens et al., 1999), the limb was randomized for both, with one device located in the preferred limb and the other in the non‐preferred limb. The preferred leg was determined by asking the question, “If you would kick a ball on a target, which leg would you use to kick the ball?” (van Melick et al., 2017). Location was determined at 2/3 of the distance between the line from the anterior spina iliac superior to the lateral side of the patella. The NIRS monitor had an inter‐optode spacing of 25 mm, and the sampling frequency was 0.5 Hz. The SmO_2_ data were filtered using a Butterworth low‐pass filter with a cut‐off frequency of 0.2 Hz (Rodriguez et al., 2018). SmO_2_ data were analyzed across the entire 40‐s period corresponding to the duration of the squat. In addition, variation of the SmO_2_ (ΔSmO_2_) was calculated as the difference between each value with the initial value of each squat.

For the analysis of muscle excitation, the Trigno Avanti device and the Trigno Galileo EMG sensor (Delsys Inc., USA) were used. Although the Galileo sensor is a high‐density EMG sensor, for this study, it was used to obtain the Root Mean Square (RMS). The data were recorded at a sampling frequency of 2222 Hz using the Trigno Discover software, which was also used for signal analysis (Delsys Inc., USA). The EMG signal was filtered with the Butterworth bandpass at 20–450 Hz of order 4 (Merletti & Cerone, 2020; Potvin & Brown, 2004). Participants were prepared before sensor placement by shaving off their skin and cleaning with alcohol. Before measurements, a maximum voluntary contraction (MVC) of the vastus lateralis was performed, which was used to normalize the RMS value. Participants were seated with the knee flexed at 90°, and the foot was placed against a rigid, immovable bar attached to a stable structure to prevent joint movement. The evaluator ensured proper stabilization of the participant and provided strong standardized verbal encouragement throughout the effort. Participants were instructed to exert maximal force for 5 s while maintaining the isometric position. Three trials were performed following the standard Trigno Discover software protocol and the highest RMS value was retained for normalization. Because the Galileo sensor provides the RMS value from its four built‐in sensors, the RMS values obtained from the four sensors were averaged. For statistical analysis, the RMS average was obtained during seconds 30 and 35 of the isometric squat, ignoring the final part of the squat, during which the participant could have made position adjustments due to fatigue.

### 2.4 Statistical analysis

Statistical analysis was performed using RStudio (version 2024.12.1) and Python (Anaconda 2.6.5) for the statistical parametric mapping (SPM) analysis (SPM1D package). The significance limit was set at *p* < 0.05. First, the normality of the variables was analyzed using the Shapiro–Wilk test, observing that all variables presented a normal distribution (*p* > 0.05). For this reason, to analyze the effect of knee flexion on RMS, a repeated measures ANOVA with Bonferroni tests for pairwise comparisons was used. When performing the ΔSmO_2_ analysis, we applied a one‐dimensional SPM to analyze the time series signal throughout the isometric squat. To do this, the three isometric squats were normalized from 0 to 100% of their duration. A repeated‐measures SPM ANOVA was performed with knee flexion angle as the within‐subject factor with three levels (80°, 90°, and 100°). When a significant main effect was detected, post hoc paired SPM *t*‐tests were conducted. Statistical inference was performed using Random Field Theory to control the family‐wise error rate across the entire time series (*α* = 0.05). For the analysis of the RMS, an ANOVA with one within‐subject factor (knee flexion) was performed with the Student's *t*‐test with Bonferroni correction as a post hoc. For significant pairs differences, Hedge's effect sizes (ESg) were computed with paired correction and classified as small (ESg 0.2–0.5), moderate (Esg 0.5–0.8), or large (ESg > 0.8) (Cohen, 1988; Hedges, 1981). Data are presented as means and standard deviations, as well as 95% confidence intervals (95% CI) for the differences between conditions.

## 3 RESULTS

### 3.1 Muscle oxygen saturation

**FIGURE 2:**
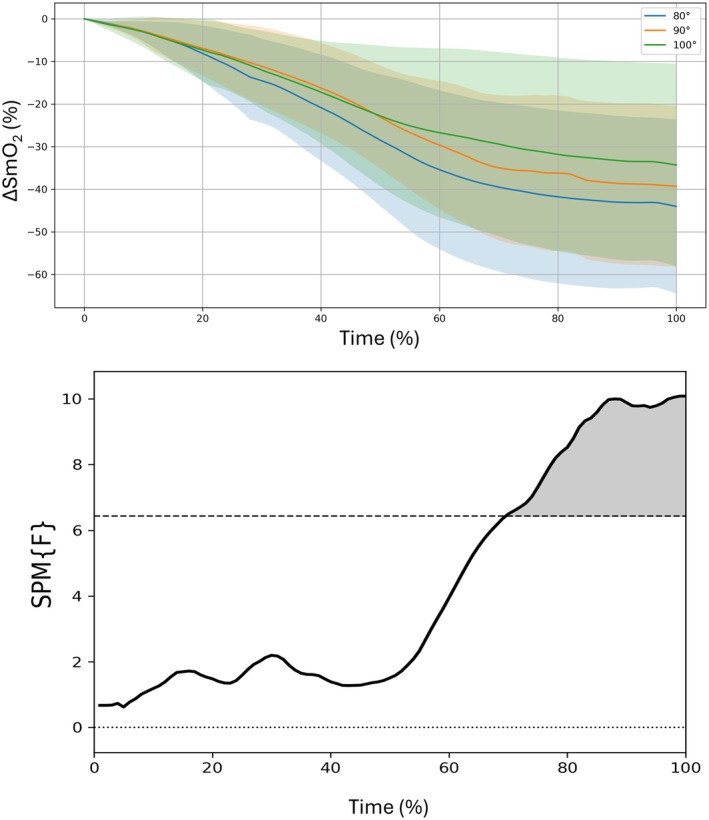
Upper panel: Mean (line) and standard deviation (shadow) of the muscle oxygen saturation (SmO_2_) response of the three knee flexion conditions during the 40‐s isometric squat. Lower panel: ANOVA statistical parametric mapping between the three conditions. The vertical axis displays the statistical value {F} and a significant effect is present at instances where the black line is above the upper horizontal dotted line.

**FIGURE 3:**
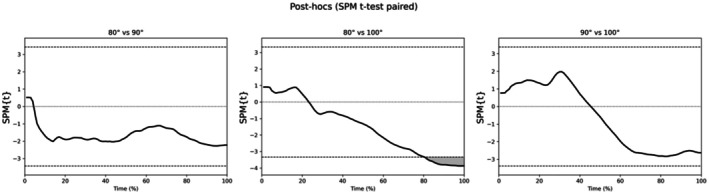
T‐tests of the statistical parametric mapping of muscle oxygen saturation between the three knee flexion conditions during the 40‐s isometric squat. The vertical axis displays the statistical value {t}, and a significant effect between pairs is present at instances where the black line is above the upper horizontal dotted line or below the lower horizontal dotted line.

Figure 2 shows the response of the ΔSmO_2_ during the 40‐s isometric squat at the three knee flexion conditions, and the ANOVA of the SPM. The ANOVA shows that there is a difference in the last third of the test. Moreover, Figure 3 shows the post hocs, when the differences observed were between the 80° and 100° conditions at the last 20% of the test. At the end of the test (100%), the mean difference between conditions was 13% (*p* < 0.01, ESg = 0.9 and 95% CI [6, 21%]). No differences were observed between the 90° and the other two knee flexion conditions (i.e., 80° and 100°).

### 3.2 Muscle excitation

**FIGURE 4:**
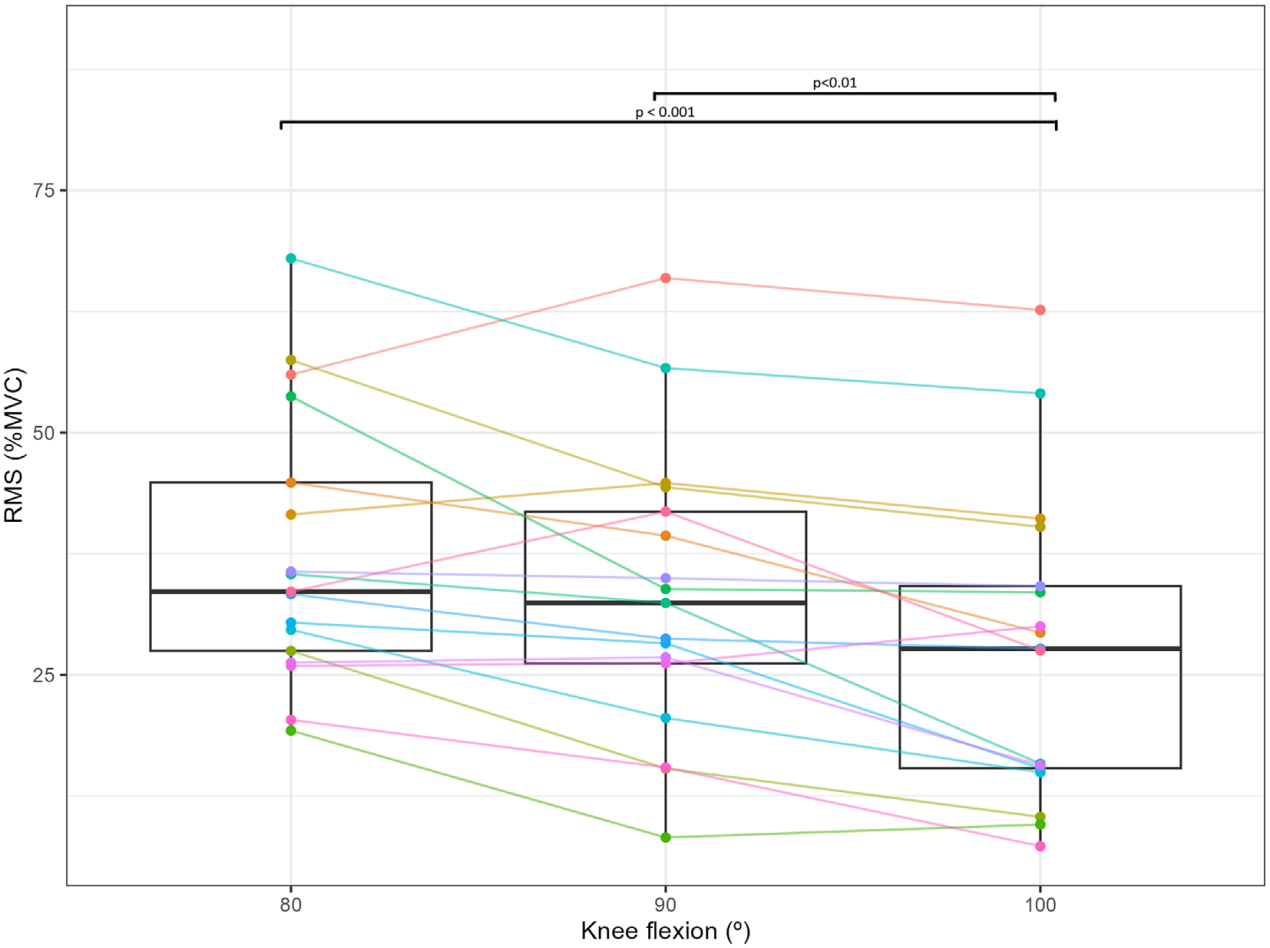
Boxplots (median, interquartile range, and whiskers to 1.5 × interquartile range) of Root Mean Square (RMS) of muscle excitation normalized by maximum voluntary contraction (MVC) during the 30‐ and 35‐s of each 40‐s isometric squat at the three knee flexions performed. Differences between conditions are shown by the *p* value. Horizontal lines join individual data.

The RMS of the three knee flexions differs between them (Figure 4). The RMS of the squat at 100° was lower than at 80° (*p* < 0.001, ESg = 1.2 and 95% CI [5.8, 14.2%]) and 90° (*p* < 0.01, ESg = 0.9 and 95% CI [2.6, 8.5%]). No differences were obtained in RMS between 80° and 90° (*p* = 0.10).

## 4 DISCUSSION

This study aimed to assess the effect of the knee flexion during a 40‐s isometric squat on the SmO_2_ response. The main results were that although the three knee flexions assessed (80°, 90°, and 100°) differed between them in muscle excitation, with lower RMS for the 100° than the other two flexions, SmO_2_ only differed between the two extreme conditions (80° and 100°), presenting a higher deoxygenation at 80° toward the end of the test.

We hypothesized that a 10° difference in knee flexion would not affect SmO_2_, while a 20° difference would yield detectable changes, consistent with differences in muscle excitation. Our hypothesis was accepted as the results showed no differences in SmO_2_ responses between the 90° and 100°, but 80° knee flexion had a higher deoxygenation at the end of the isometric squat compared to 100°. Differences in SmO_2_ emerged only during the final 20% of the task, likely because deoxygenation during sustained isometric contractions develops progressively as oxygen delivery becomes increasingly constrained while metabolic demand is maintained, leading to clearer separation between conditions toward the end of the contraction (Barstow, 2019; de Ruiter et al., 2005). Higher‐demand muscular actions are associated with higher deoxygenation because of the highest demand and availability of oxygen (Song et al., 2025). This is supported by the lower muscular excitation observed at 100° compared with the other knee flexions. Therefore, our results are in agreement with previous literature showing a certain relationship between muscle excitation and oxygenation responses both in cyclic sports (i.e., cycling) (Iannetta et al., 2017; Sendra‐Pérez, Encarnación‐Martínez, et al., 2025) and in strength exercises (Song et al., 2025; Taelman et al., 2011).

From a biomechanical perspective, small angular variations (10°) around 90° may not substantially modify quadriceps torque production, as peak joint torque typically varies gradually within this midrange of knee flexion (Anderson et al., 2007). In contrast, a 20° difference may shift the muscle to a distinct region of the length–tension relationship and alter joint moment arm characteristics (Herzog & Read, 1993), potentially increasing mechanical demand and muscle recruitment (Marchetti et al., 2016). Metabolically, greater muscle excitation and intramuscular pressure during sustained contractions may increase oxygen extraction and limit local perfusion, leading to more pronounced SmO_2_ desaturation (Barstow, 2019; Scano et al., 2020; Sendra‐Pérez, Encarnación‐Martínez, et al., 2025).

The integration of EMG and NIRS into a single portable sensor system has gained increasing interest in exercise physiology and rehabilitation, as it enables simultaneous and complementary information about muscle fatigue, since it shows an electrical and metabolic perspective (Guo et al., 2022; Kimoto, Fujiyama, & Machida, 2023; Kimoto, Oishi, & Machida, 2023; Lomeli‐Garcia et al., 2023; Maliszewski et al., 2024). Although the present study employed separate EMG and NIRS devices, the results can provide some insights into the combination of both. EMG provides high‐temporal resolution information on muscular excitation by recording electrical potentials associated with muscle contractions, whereas NIRS technology quantifies the muscle oxygenation by tracking changes in oxy‐ and deoxyhemoglobin concentrations during exercise (Kimoto, Oishi, & Machida, 2023). Importantly, these modalities differ substantially in sampling frequency and physiological response characteristics. EMG captures rapid changes in neural drive, while NIRS‐derived SmO_2_ reflects slower hemodynamic adjustments and cumulative metabolic stress during sustained contractions. The combination of these modalities in a unified device not only reduces the physical interference of multiple sensors on the body, but also enhances the physiological breadth of muscle fatigue monitoring (Kimoto, Fujiyama, & Machida, 2023). Recent advances in wireless multi‐modal sensor systems have demonstrated their utility in dynamic exercises such as cycling, allowing for more precise anaerobic threshold estimation through joint analysis of EMG and NIRS signals (Kimoto, Oishi, & Machida, 2023). In our study, EMG appeared more sensitive in detecting immediate condition‐specific differences, whereas NIRS differences emerged toward the end of the isometric task, likely reflecting progressive alterations in oxygen extraction rather than instantaneous neuromuscular changes. However, the continuous nature of NIRS measurement (more straightforward than EMG) revealed that condition‐related differences emerged particularly toward the end of the isometric task, suggesting that NIRS may be more informative for capturing cumulative metabolic stress during prolonged muscle excitation (Maliszewski et al., 2024).

Our findings suggest that 90° of knee flexion represents an intermediate point in terms of both metabolic and neuromuscular demand during a 40‐s isometric squat. Although Marchetti et al. (2016) reported that 90° is the most effective position during an isometric squat considering torque and muscular excitation, our results contrast with the study of Trindade et al. (2020) which shows a higher muscular excitation at 90° than at 60°, and the study of Kukić et al. (2022) that showed no differences in muscular excitation in knee flexions between 80° and 130°. One possible explanation of the differences observed is the experimental settings. While these previous studies performed their squats in short times (5‐s), our squats were performed for 40‐s. This duration was chosen to better reflect field testing conditions, particularly when the aim is to assess fatigue status, a phenomenon that typically manifests toward the latter stages of sustained efforts. This rationale is supported by our results, which showed that SmO_2_ differences between conditions only emerged in the final 20% of the test.

The results of the present study have important applications for evaluating whether a 40‐s isometric squat could be a useful test for determining fatigue status using NIRS technology in field. First, it is observed that the 90° knee flexion position appears to be a good posture as it has an intermediate metabolic demand and muscle excitation. Furthermore, the fact that only 20° differences in SmO_2_ response were detected between conditions may indicate that ensuring correct posture is important, but in the field, it may not be necessary to record test performance using motion analysis, and the use of a goniometer alone may be valid, considering accessibility and acquisition time (Canever et al., 2025; Lind et al., 2022). Finally, the results also demonstrate that 40‐s of this type of test is sufficient to detect differences between conditions with different demands. However, very short tests would not allow for detecting differences as these occurred in the last 20% of the test (i.e., in the last 8‐s).

This study is limited by its sample size, which was not powered to analyze differences based on sex or adipose tissue thickness, two factors known to affect NIRS accuracy (Ansdell et al., 2019; Niemeijer et al., 2017). Additionally, as the sample consisted of physically active individuals, the findings should be interpreted within this context and may not necessarily generalize to sedentary, older, or clinical populations. Future research should examine the inter‐day reliability of SmO_2_ responses during 40‐s isometric squat, as well as how variables such as prior exercise, fatigue status, or muscle damage influence the response.

## 5 CONCLUSIONS

Our results suggest that a knee flexion of 90° during an isometric squat elicits intermediate levels of muscle excitation and oxygenation_2_ response, and that a 40‐s contraction is sufficient to reveal condition‐specific differences. A minimum difference of 20° in knee flexion appears necessary to elicit changes in SmO_2_, supporting the practical use of goniometers as a viable tool for posture control in field‐based muscle monitoring.

## FUNDING INFORMATION

The project was funded by Conselleria de Innovación, Universidades, Ciencia y Sociedad Digital of Generalitat Valenciana (Ref: CIGE/2023/80). JMM‐F’s contribution was funded by a pre‐doctoral grant from the Ministry of Universities of Spain (ref FPU20/01060).

## CONFLICT OF INTEREST STATEMENT

The authors declare no conflicts of interest.

## ETHICS STATEMENT

The Ethics Committee of the University of Valencia guaranteed the ethical approval to carry out this project with the document registered under number IRB 2024‐FIS‐3694985.

## Data Availability

The dataset used for the analysis of this work is in a public repository (Mendeley data; doi: 10.17632/h6v35pkfr3.1).
